# Are women breaking the glass ceiling? A gendered analysis of the duration of sick leave in Spain

**DOI:** 10.1007/s10754-023-09351-2

**Published:** 2023-04-24

**Authors:** Ángel L. Martín-Román, Alfonso Moral, Sara Pinillos-Franco

**Affiliations:** 1 Dpto. Análisis Económico, Facultad de Ciencias Sociales Jurídicas Y de La Comunicación, Universidad de Valladolid, Plaza Universidad, 40005 Segovia, Spain; 2 Dpto. Análisis Económico, Facultad de Ciencias Económicas Y Empresariales, Universidad Autónoma de Madrid, Calle Francisco Tomás Y Valiente 5, 28049 Madrid, Spain

**Keywords:** Moral hazard, Glass ceiling, Workplace injuries, Gender, Stochastic frontiers, I12, I13, I18, J28

## Abstract

We study the gender gap in the duration of sick leave in Spain by splitting this duration into two types of days – those which are related to biological characteristics and those derived from behavioral reasons. Using the Statistics of Accidents at Work for 2011–2019, we found that women presented longer standard durations (i.e., purely attached to physiological reasons) compared to men. However, when estimating individuals’ efficiency as the ratio between actual and standard durations, we found that women were more inefficient at lower levels of income, whereas in case of men, this occurred at higher levels of income. These results were reinforced when considering that men and women do not recover from the same injury at the same rate. Women were more efficient than men across all the compensation distribution, especially at higher income levels.

## Introduction

Economic research is key to understanding sickness absence and its connection with the labor market (Boone & van Ours, 2006; Boone et al., 2011; Pichler, 2015). Workplace injuries have cost the European Union over EUR 460 billion in 2019 (European Commission, 2021), and behavioral effects play an important role in explaining this cost (Koning et al., 2022; Martin-Roman & Moral, 2016; Martín-Román & Moral, 2017). Despite these figures, the literature examining gender/sex differences in the duration of sick leave is lacking, considering the behavioral response of workers to a set of incentives and focusing on biomedical ex-post (after the injury has been reported) differences between male and female workers. Therefore, from a policy-making perspective, understanding the main drivers behind those differences is important to better implement measures aimed at enhancing social welfare.

This study aims to fill this gap by studying the durations of sick leave in men and women related to workplace accidents (hereafter, we will refer to it as “sick leave”). It is also worth mentioning that our main interest is not only measuring the differences between both sexes but also identifying the underlying physiological factors behind them. Moreover, we intend to measure differences as a result of different choices (i.e., behavioral responses) made by men and women, which lead to different outcomes.^1^ Finally, we check whether these differences are the same at the top and bottom of the income distribution ladder, as the behavior of each individual is influenced by different economic incentives such as sick pay (Koning et al., 2022; Puhani & Sonderhof, 2010), salary (Brekke et al., 2020), or income in general (Ásgeirsdóttir & Jóhannsdóttir, 2017). Thus, the research questions of this study can be stated as follows:

1. Are there differences in the sick leave durations between male and female employees as a result of a workplace injury? If so, are these differences a consequence of biomedical traits or behavioral responses?
2. Are these differences constant across income percentile levels?

To answer the abovementioned research questions, we used the stochastic frontier methodology. This technique allows us to divide the total sick leave duration into two different parts: one associated with physiological factors and one related to behavioral responses of workers to socioeconomic incentives (Martín-Román & Moral, 2014, 2017). We draw the main conclusions obtained in this research by analyzing both components.

The results show that if we assume that men and women share the same biological characteristics (i.e., they recover at the same rate after an injury or illness), the behavioral responses were similar between the two groups when looking at the whole distribution.. However, our findings changed when considering statistically the existence of physiological differences. Overall, we observed that women had poorer health than men, resulting in longer physiological recovery periods than them for the same type of injury, severity, and other biological determinants. In turn, this shows that women exhibit less opportunistic behavior in general terms.

When looking at wage distribution, significant outcomes emerged. As the wage rises, different patterns of opportunistic behavior between men and women occur. The opportunistic behavior in men increased with wage monotonically, whereas in women such opportunistic behavior increased first, but after reaching a relative maximum, their behavioral response substantially declined in the upper part of the wage distribution level. Because of these patterns, the gender gap in the sick leave duration is of a significant size for high wage levels. Hence, the developed empirical evidence follows the observed gender differences in the labor market of Western economies, where women tend to face a glass ceiling in their professional advancement, and attempt to break it by trying harder than men.

This study includes valuable evidence to the literature that covers gender differences in the workplace. In particular, using a stochastic frontier approach, we clearly distinguished the physiological factors from behavioral factors for the duration of sick leave. This approach, to the best of our knowledge, implies a twofold novelty—a methodological advance and further evidence on the differences in potential opportunistic behavior between men and women.

Additionally, we also tested whether behavioral differences between men and women were constant across the wage distribution to better understand the whole phenomenon. This allowed us to check whether injured women behaviorally react to *break* the glass ceiling. Applying the theories of compensating wage differentials and efficiency wages, some firms may be willing to tolerate some level of shirking in exchange for lower wages or offer higher wages to reduce the probability of absenteeism (Suárez & Muñiz, 2018).

From a policy standpoint, the results of this study are valuable in designing well-targeted measures. On the one hand, policymakers may identify to what extent the length of sick leave is due to physiological or behavioral issues and how these two components affect each gender or sex differently. This evidence-based knowledge can lead to an improved public health policy design, which will help policymakers to decide whether to devote more financial resources to workplace safety instruments or to monitor activities aimed at reducing opportunistic behaviors. On the other hand, the results can also serve as the basis for implementing antidiscrimination policies, pointing toward different behavioral responses between men and women at the upper part of the wage distribution level.

The rest of the paper is organized as follows. Section two presents and discusses the literature related to health differences between men and women at work. In section three, we review the disability and work-related accident schemes in Spain. Sections four and five describe the database and accounts for the methodological approach employed in this research. Section six shows the main results obtained. Section seven includes a robustness check. Section eight discusses such results, emphasizing the policy implications, and provides some recommendations. Finally, section nine concludes.

## Sex, gender, and sick leave

The duration of sick leave is a gender- and sex-dependent phenomenon. In general, it is well known that women tend to extend their recovery periods longer than men (Fontaneda et al., 2019; Moral de Blas et al., 2012) and there exist some hypotheses that explain this situation (Østby et al., 2018).

For example, women are more involved in domestic chores than men (such as cooking, cleaning, childcare.),^2^ implying a double burden and higher depression levels when they are employed (Pinquart & Sörensen, 2006), leading to longer recovery periods.

Sex differences in labor participation also play a role in explaining the gender/sex gap in the duration of sick leave. The percentage of women actively working in Spain is lower compared with that of men,^3^ and they are also more engaged in precarious jobs^4^ (Franco & Winqvist, 2002; Kim et al., 2008; Menéndez et al., 2007), which leads to high stress levels and insecurity, which negatively affects their health (Rugulies et al., 2008).

Additionally, men and women do not present the same prevalence of illnesses. Women are more prone to present conditions that disable them on a daily basis, such as rheumatism, anemia, thyroid, eczema, headaches, or mental illnesses, whereas men present high odds of fatal illnesses, such as cardiovascular diseases, stroke, lung and kidney diseases, or liver cirrhosis (Case & Paxson, 2005; Macintyre et al., 1996). Women also tend to present higher rates of work disability, that is, physical or psychological symptoms that do not necessarily prevent them from returning to work but complicate their recovery return after their sick leave (Coutu et al., 2021).

Therefore, it is important to distinguish between sex and gender when tackling differences in the duration of sick leave. Sex refers to the physical and biological characteristics that define a male or female, which includes hormonal and anatomic differences, whereas gender refers to the roles, behaviors, and personal identity (Bechtel & Huffmyer, 2020).

Biological differences between men and women may partly explain why women present longer sick leave durations. Women are more likely to suffer from musculoskeletal diseases compared with men,^5^ which leads them to require longer recovery periods after injury and surgery (Wolf et al., 2015). Because of reproductive biology and conditions specific to gender, they use more healthcare services than men (Kananurak, 2014). Women are also more prone to return to hospital after a cardiac surgery compared with their male counterparts because they have smaller coronary arteries and lower surface area, which complicates the efficacy of a bypass and grafts (Bechtel & Huffmyer, 2020). Moreover, women tend to present higher odds of dysphoria, anxiety, and depression after these types of surgical interventions or traumatic events, thus expanding their sick leave durations (Freedman et al., 2002; Kempen et al., 2003; Modica et al., 2014; Oksuzyan et al., 2018).

From a gender viewpoint, there still exists a traditional role attached to women that may lead them to extend their return to work (Côté & Coutu, 2010), as stated at the beginning of this section. However, there are also gender differences in behavior/preferences (Schünemann et al., 2017) affecting the health of each individual (Bauer et al., 2007; Nelson, 2014), accounting for the gender gap in sick leave duration as well. For example, women are more risk averse, less competitive, and more context-sensitive in making their decisions than men (Croson & Gneezy, 2009). Additionally, women present higher social risk aversion (i.e., when individual decisions influence not only the individual’s payoffs but also the payoffs of another individual) and higher inequality aversion compared with their male counterparts (Friedl et al., 2020).

These gender differences in behavior were also tested during the COVID-19 pandemic. Women were more likely to accomplish with the public measures imposed by the different governments than men (Galasso et al., 2020). This may be because women are more aware about their health status than men (Idler, 2003), which leads them to adopt “cautious behaviors” when returning to work after their sick leave.

## Sick leave benefits in Spain

In Spain, when a worker is temporarily unable to work as a result of an illness or an accident, whether work-related or non-work-related, the Social Security Administration covers the medical expenses of the injured worker, as well as compensates the loss of income during his/her temporary incapacity (TI). The maximum duration of the TI is 365 days, potentially extendable 180 days more only if the worker is presumed to recover from his/her incapacity during this extension.^6^ After this period, the National Institute of Social Security is the competent authority to determine whether the worker receives a medical discharge, or is transferred to the permanent disability system.

**Fig. 1:**
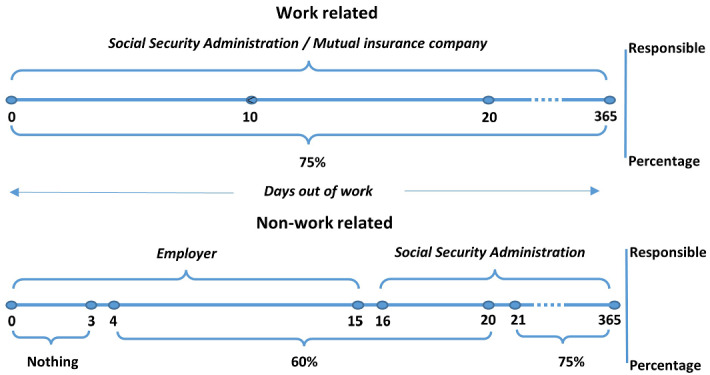
TI benefits. Source: Own elaboration

The TI benefits consist of a subsidy equivalent to a percentage of the contributory base, which is mainly the wage earned during the previous months. However, depending on whether the TI was caused by a common illness or by a working accident, or a professional illness, the rules of receiving the TI benefits change. If the TI is due to a common illness (i.e., non-work-related illness or injury), during the first three days of sick leave the worker does not receive any amount of sick leave. From the 4th and 20th day the worker receives the 60% of the reference wage (i.e., the previous month's wage), and finally, he/she will receive the 75% from the 21st day onwards. The employer has to pay the injured worker from the 4th to the 15th day, and from the 16th day onwards, the Social Security Administration assumes this payment. On the contrary, when the TI was caused by a working accident or professional illness, the worker receives the 75% of the reference wage from the day after the general practitioner issues the sick leave certificate, and this is paid by the mutual insurance company (see Fig. 1).

For private sector employees, the law that regulates sick leave benefits is the Social Security General Law (SSGL) of 1994, and the rules affecting them have been unchanged since then. In the case of public sector workers, the law changed in 2012 in an attempt to limit public expenditure. The interested reader can find a recent study that explored the impact of this legal change on work absence in Spain (Marie & Vall Castello, 2022). In this paper we focus our attention on private sector workers who are on TI due to a working accident, therefore, no law change has occurred during the period analysis of our study. Article 156 of the SSGL defines a working accident as any bodily injury suffered by a worker that occurred because of executing paid employment.^7^

Regarding contributory base, this is estimated as a function of the previous month's wage (with upper and lower limits) earned by the worker before the working accident. Upper limits of the contributory base are yearly established in the State General budget and they are the same for all professional categories and contingencies (Art. 148 of the SSGL). In the case of lower limits of the contributory base (Art. 19.2 of the SSGL), the amount corresponds to the current minimum wage of each year, increased by one-sixth, unless expressly provided otherwise. The contributory base has not changed during the period analysis of our study either.

## Database

In this study, the dependent variable is the sick leave duration resulting from a workplace accident. As a result, the database offering the best information is the Statistics of Accidents at Work (SAW) published by the Spanish Ministry of Labor. This is an annual register of any occupational accident resulting in the inability of an individual to work in Spain. Moreover, this database provides information regarding the characteristics of the injured person, enterprise, workplace, accident, and information about the nature and seriousness of the injuries.

**Fig. 2:**
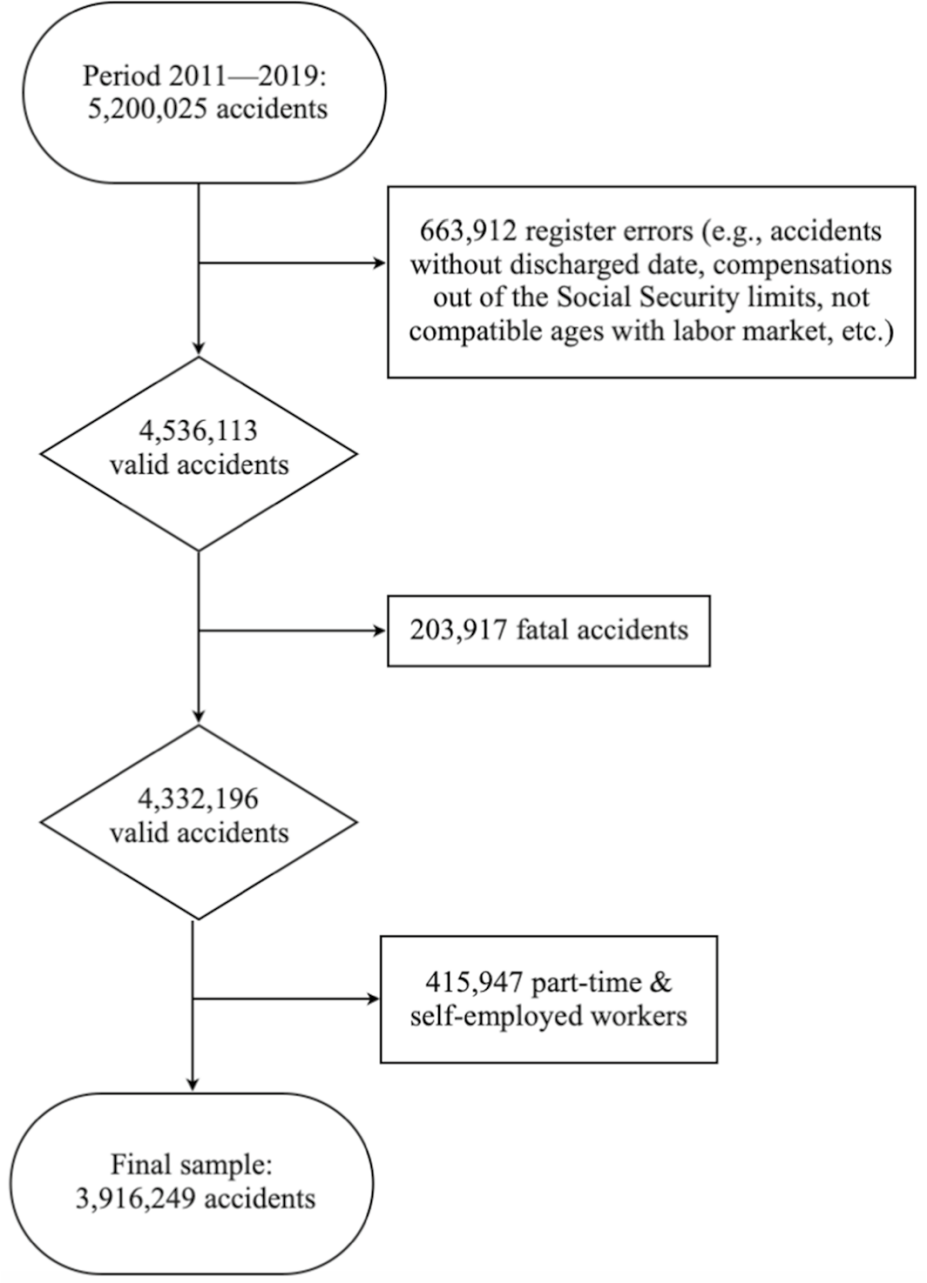
Data cleansing flow chart. Source: Own elaboration

In this study, we used the 2011–2019 waves of the SAW. Figure 2 shows the detailed flow chart of the data cleansing process. We first filtered this database to eliminate register errors and to obtain homogeneous information that allows us to detect the causal effects we are interested in. Given that we aim to analyze the behavior of workers according to the economic compensation received when they are on leave, the compensation variable was the first one used to filter the sample. Thus, all those accidents that reported a compensation outside the limits registered by the Social Security were eliminated, and the existing inflation in the period was corrected.^8^ This procedure does not imply that we have removed accidents of the high contributory base, but all those accidents that are attached to compensations that are not legally possible. We also excluded registration errors referring to nonexistent contracts or ages incompatible with the labor market. In the next step, data on fatal accidents were omitted because after these types of accidents, it is not possible to detect any worker’s behavior. Finally, part-time workers were eliminated because they might affect the values of the daily compensation received and the self-employed were also excluded because they follow a different incentive scheme compared with employees. Evidence from a Dutch sample suggested that their sick leave durations are not affected by moral hazard when considering the level of economic compensation (Spierdijk et al., 2009). After this data cleansing, the final database comprised 3,916,249 records of occupational accidents with sick leave.

Table 1 gives an initial review of the data regarding the length of each period of sick leave broken down into the following: the sex of the worker, the percentage of males, and the number of observations by different characteristics. Aggregate figures show that 72% of injured workers are male and that the average duration is 0.8 days longer for women. There is also a gradual increase in the duration over time. The sick leave duration among women increased by 4.5 days from 2011 to 2019, whereas in men, it was 3.5 days longer over the period analyzed.

**Table 1:** Mean durations by gender, percentage of males and number of injuries in terms of various characteristics. Source: Author’s own based on SAW data

	Duration (days out of work)		% Male	Data
	Female	Male		
*Type of injury*				
Not specified	30.84	28.04	69.0%	73,802
Superficial Injuries	25.13	21.64	73.1%	629,517
Other injuries	23.07	22.84	79.5%	615,733
Fractures	79.09	74.10	75.0%	286,993
Strains	30.94	29.50	68.9%	637,301
Dislocations	33.20	33.23	70.6%	276,511
Sprain	29.84	29.45	67.7%	1,017,476
Traumatic amputation	80.22	88.37	89.2%	9,110
Concussion	31.95	33.45	73.4%	223,090
Burns	15.47	20.30	73.5%	50,961
Poisoning	17.03	17.27	62.6%	6,159
Choking	21.94	21.41	62.5%	2,017
Noise, heat	19.91	22.51	75.0%	5,737
Psychological trauma	41.57	35.49	64.5%	13,330
Multiple injuries	40.76	49.74	68.6%	62,723
Heart attack	136.22	164.46	85.9%	5,789
*Year of the injury*^a^				
2011	29.46	29.39	73.5%	462,734
2012	29.72	29.72	72.0%	362,463
2013	31.93	31.66	70.4%	348,032
2014	31.66	31.26	70.1%	394,384
2015	32.78	31.92	70.7%	420,347
2016	32.77	31.59	70.8%	445,308
2017	33.29	32.40	72.2%	478,551
2018	33.85	32.20	72.7%	494,338
2019	33.96	32.75	73.4%	509,435
*Workers compensation percentile*				
p0–p5	29.93	28.99	58.4%	195,764
p5–p10	30.11	29.82	56.9%	195,858
p10–p25	30.37	29.87	62.4%	587,347
p25–p50	31.62	30.82	74.3%	979,249
p50–p75	33.18	31.44	75.0%	978,964
p75–p90	34.72	32.58	77.4%	587,394
p90–p95	35.80	33.74	78.6%	195,499
p95–p100	38.42	36.60	77.6%	195,874

	Duration (days out of work)		% Male	Data
	Female	Male		
*Occupation*				
Company management	37.84	41.72	64.4%	13,791
Technical staff and scientists	34.97	36.58	31.0%	144,724
Professional support	32.76	34.98	66.9%	143,180
Administration employees	34.82	36.24	36.1%	188,773
Service workers	31.05	30.99	48.5%	795,967
Skilled agriculture and fishing	31.99	33.13	88.4%	100,537
Crafts and dealers	32.64	31.04	93.8%	947,978
Machine operators	33.18	33.89	93.1%	548,405
Unskilled	31.72	29.10	70.0%	1,032,894
*Sector of activity*				
Agriculture, fores-try and fishing	31.63	33.18	82.4%	230,928
Energy and Water	33.25	32.31	87.1%	78,021
Minery	52.43	39.73	97.7%	20,905
Manufacturing I	33.62	30.82	75.4%	247,448
Manufacturing II	31.40	30.11	91.0%	497,438
Construction	33.34	32.96	98.1%	497,474
Wholesale and retail trade; repair of motor vehicles, accommodation, food services…	29.75	28.56	61.9%	846,941
Transportation and comunication	31.97	34.76	83.0%	306,176
Finances, insurance activities…	30.30	28.61	66.4%	222,132
Other services	34.20	32.82	49.8%	968,786
Total	32.28	31.50	71.9%	3,916,249

^**a**^There are 657 work place accidents that took place in 2010

Following the compensation variable, some interesting results were also observed. As the amount of compensation rose, the duration of sick leave was longer. Moreover, the percentage of men in higher percentile levels was wider than that of women. Finally, we observe an increase in the difference in duration between men and women as the amount of compensation rises. Under the median, the sick leave duration of women exceeded that of men in less than 1 day (0.3 days between percentiles 5 and 10). However, over percentile 50, this difference was over 2 days.

## Methods

Different estimation techniques were applied to the empirical literature analyzing the duration of accident-related sick leave. Some authors used simple regression techniques, such as ordinary least squares (OLS) (Campolieti & Hyatt, 2006; Krueger, 1990) or hazard models (Corrales Herrero et al., 2008; Moral de Blas et al., 2012; Murcia López et al., 2016). Other works used the days out of work as a count variable and applied count data models (Delgado & Kniesner, 1997; Frick & Malo, 2008; Lechmann & Schnabel, 2014) or zero-inflated negative binomial models (Mukuria et al., 2017). There are also some studies that gauged on how changes in legislation affected sick leave duration using natural experiments (Goerke & Pannenberg, 2015; Meyer et al., 1995; Ziebarth & Karlsson, 2010). Fortin and Lanoie (2000) presented a detailed discussion on the different estimation methods within this context.

This paper adopts a different and more recent approach based on the stochastic frontier analysis. Although this type of analysis is more common in papers related to production or cost theory, there already exist previous studies that applied it to the duration of sick leave (Martín-Román & Moral, 2014, 2017). In this case, the variable of interest is the days a worker is absent from work due to an occupational accident. These absence days are considered a cost determined by medical variables such as the type of injury suffered, its severity, the injured part of the body, and other factors such as individuals’ age or gender if we consider physiological differences between men and women. All these variables form the inputs of our function. With this approach, the efficient durations (i.e., cost efficiency) would correspond to those injured workers who present a shorter recovery period in similar circumstances (inputs). This similarity covers presenting the same physiological characteristics (age and sex), suffering from the same injury with the same severity, and that affects the same part of the body. The distance between that minimum duration and the actual duration of sick leave determines the degree of inefficiency related to the recovery process of injured workers.

From this interpretation of the stochastic frontiers, this methodology allows us to obtain a lower boundary attributed to medical and physiological factors labeled as “standard sick leave duration” ${\text{(D}}_{\rm i}^{{\rm s}}{)}$. This minimum recovery spell required to return to work is defined by the following expression:

1 $${\text{d}}_{{\rm i}}^{{\rm s}} = {\text{X}}_{{\rm i}}\beta + {\text{v}}_{{\rm i}},$$

where ${\text{d}}_{{\rm i}}^{{\rm s}}$ is the logarithm of the minimum duration (${{\text{d}}_{{\rm i}}^{{\rm s}}{ = ln(D}}_{{\rm i}}^{{\rm s}}{)}$), $\beta$ is a vector of coefficients, ${\text{X}}_{{\rm i}}$ is a vector of individual characteristics, and ${\text{v}}_{{\rm i}}$ is a random error of mean *zero* and variance ${\sigma}_{\text{v}}^{2}$*.*

However, there is an information problem that prevents the minimum duration being known. The insurer only perceives the actual duration ${\text{(D}}^{{\rm r}}{)}$, which tends to be longer than the standard duration ${\text{(D}}^{{\rm r}} \, \geq \, {\text{D}}^{{\rm s}}{)}$. In addition to medical and physiological aspects,${\text{D}}^{{\rm r}}$ is the result of the ability of the workers to obtain an additional period of recovery. This ability reflects an opportunistic behavior on the part of the worker, which indicates how secure they perceive their job situation.^9^ Obviously, greater job security would be linked to better working conditions, which enables the worker to engage more intensely in such opportunistic behaviors. This leads us to conclude that we face a problem of asymmetric information that insurance companies assume when monitoring workers during their work absence.

Therefore, there is a real duration that is the result of adding the standard duration to a non-negative random disturbance. As explained previously, the standard duration forms a lower frontier^10^ that may be estimated based on physiological factors, such as age or the nature of the injury, and that determines the length of recovery time.

2 $${\text{d}}_{{\rm i}}^{{\rm r}} = {\text{d}}_{{\rm i}}^{{\rm s}} + {\text{u}}_{{\rm i}},$$

where $${\text{d}}_{{\rm i}}^{{\rm r}}$$ is the logarithm of the actual duration $${({\text{d}}_{{\rm i}}^{{\rm r}} = \text{ln}({\text{D}}}_{{\rm i}}^{{\rm r}}$$,$${)}{)}$$ and $${\text{u}}_{{\rm i}}$$ is another error term with a positive mean and variance $${\sigma}_{\text{u}}^{2}$$. $${\text{u}}_{{\rm i}}$$ must follow a certain statistical distribution to ensure that the model may be estimated. The literature offers several examples of distributions, such as half-normal (Aigner et al., 1977), exponential (Meeusen & van Den Broeck, 1977), truncated normal (Stevenson, 1980), or gamma (Greene, 1980a, 1980b). We decided to use exponential distribution following Meeusen and van Den Broeck (1977). Following (1) and (2), we obtain the following expression:

3 $${\text{d}}_{{\rm i}}^{{\rm r}} = {\text{X}}_{{\rm i}}{\beta} + {\text{v}}_{{\rm i}}+{\text{u}}_{{\rm i}}$$

Given that it comprises an error model and the disturbances and regressors are independent, performing an OLS regression would provide unbiased, consistent, and efficient estimators among linear regressions. However, there is inconsistency in the constant term and variances of both disturbances cannot be separated.^11^ Therefore, when using stochastic frontier technique, it is more accurate to use maximum likelihood estimations than OLS.

Additionally, it is possible to calculate the cost efficiency value of each individual using stochastic frontier analysis with the following expression:

4 $$\text{EF} = \frac{{\text{f}}\left({\text{X}}_{{\rm i}}{\beta}\right)\text{exp}({\text{v}}_{{\rm i}}+{\text{u}}_{{\rm i}}{)}}{{\text{f}}\left({\text{X}}_{{\rm i}}{\beta}\right)\text{exp}({\text{v}}_{{\rm i}}{)}}= \text{exp} {(}{\text{u}}_{{\rm i}}{)}{.}$$

This efficiency measure is used in this study as an indicator of an opportunistic behavior.

## Results

According to what was explained in the Methods section, a cost frontier implies a minimum duration for the target variable. In this concrete case, the estimated frontier represents the minimum duration of sick leave; thus, we only included the variables related to medical and physiological characteristics. These variables included the type of injury; the injured part of the body; age; whether the injured worker was initially treated in a hospital (hospital care); and whether the injury required hospitalization, was serious, or was a recurrence of a previous injury. We also regarded sex because the biological differences between men and women may explain differences in longer sick leave durations. In this case, we obtained different frontiers and recovery periods for the same injuries for men and women alike. To test the possible biological differences between men and women, we conducted some estimations by including interaction terms between the sex variable and each type of injury. This analysis allows us to know whether men and women present different recovery periods for the same injury. Results show that women experience longer sick leave durations for most injuries (Table 7 of the ″Appendix”). Men only present longer sick leave durations in five of the sixteen considered injuries,^12^ suggesting they generally have a better health status than females.

Finally, occupational controls were included because not all jobs required the same level of recovery. Manual work necessarily requires the injured part of the body to be fully recovered to assure a proper return to work, whereas in the case of nonmanual work, this aspect is not essential; for example, a professor can perform his or her work with an arm or leg put in a cast, whereas this would not be the case for a construction worker or a waiter.

We conducted four different specifications to obtain the frontier indicating the minimum duration of sick leave (Table 2). Specification I only included the abovementioned variables, Specification II incorporated individuals’ occupation, Specification III changed Specification I by including individuals’ sex, and Specification IV includes individuals’ sex and occupation. Before the results analysis, it is important to check the existence of inefficiency. To do so, we performed a maximum likelihood test, wherein the null hypothesis was that the variance of the inefficiency term takes a value of zero. If the null hypothesis was true, the stochastic frontier model reduced to an OLS model with normal errors.^13^ In our case, the test rejected the notion that the variance of the disturbance-measuring inefficiency was zero; therefore, in all cases, the stochastic cost frontier was at a 1% level of significance.

**Table 2:** Frontier estimations of the logarithm of the sick leave duration by specification. Source: Own elaboration based on SAW data

	Not including sex variable				Including sex variable			
	Specification I		Specification II		Specification III		Specification IV	
Log duration	Coef	P > z	Coef	P > z	Coef	P > z	Coef	P > z
*Injury: Ref. unspecified injuries*								
Superficial injuries	− 0.127	0.000	− 0.128	0.000	− 0.126	0.000	− 0.127	0.000
Other injuries	− 0.089	0.000	− 0.089	0.000	− 0.085	0.000	− 0.086	0.000
Fractures	1.106	0.000	1.105	0.000	1.109	0.000	1.109	0.000
Strains	0.039	0.000	0.038	0.000	0.040	0.000	0.038	0.000
Dislocations	0.100	0.000	0.099	0.000	0.101	0.000	0.099	0.000
Sprains	0.050	0.000	0.048	0.000	0.050	0.000	0.048	0.000
Traumatic amputation	1.086	0.000	1.087	0.000	1.094	0.000	1.091	0.000
Concussion	0.071	0.000	0.070	0.000	0.073	0.000	0.071	0.000
Burns	− 0.209	0.000	− 0.212	0.000	− 0.209	0.000	− 0.211	0.000
Poisoning	− 0.399	0.000	− 0.404	0.000	− 0.403	0.000	− 0.406	0.000
Choking	− 0.652	0.000	− 0.654	0.000	− 0.656	0.000	− 0.657	0.000
Noise, heat, or radiation	− 0.159	0.000	− 0.161	0.000	− 0.159	0.000	− 0.161	0.000
Psychological trauma	0.027	0.005	0.025	0.008	0.025	0.008	0.024	0.010
Multiple injuries	0.220	0.000	0.219	0.000	0.222	0.000	0.222	0.000
Heart attack	0.984	0.000	0.980	0.000	0.989	0.000	0.988	0.000
Part of the body	*Yes*		*Yes*		*Yes*		*Yes*	
*Occupation: Ref. Company management*								
Technical staff and scientists			0.017	0.052			-0.002	0.853
Professional support			0.022	0.013			0.023	0.009
Administration employees			− 0.011	0.201			-0.027	0.002
Service workers			0.027	0.002			0.017	0.041
Skilled agriculture and fishing			0.068	0.000			0.081	0.000
Crafts and dealers			− 0.009	0.274			0.006	0.447
Machine operators			0.025	0.003			0.041	0.000
Unskilled			0.002	0.783			0.005	0.567
Hospital care	0.187	0.000	0.186	0.000	0.186	0.000	0.186	0.000
Hospitalization	0.609	0.000	0.609	0.000	0.613	0.000	0.612	0.000
Serious	1.030	0.000	1.031	0.000	1.034	0.000	1.033	0.000
Recurrence	0.400	0.000	0.400	0.000	0.399	0.000	0.399	0.000
Age	0.009	0.000	0.009	0.000	0.009	0.000	0.009	0.000
Age squared	0.000	0.000	0.000	0.000	0.000	0.000	0.000	0.000
Male					− 0.050	0.000	− 0.057	0.000
Constant	1.799	0.000	1.791	0.000	1.824	0.000	1.825	0.000
sigma_v	0.856		0.855		0.855		0.855	
sigma_u	0.518		0.518		0.518		0.519	
sigma2	1.000		1.000		1.000		1.000	
Lambda	0.605		0.606		0.606		0.607	
Data	3,916,249							
LR test of sigma_u = 0: chibar2(01) = 3.4e + 04 Prob ≥ chibar2 = 0.000								

Across all specifications, the longest standard durations corresponded to accidents that led to fractures, traumatic amputation, and heart attacks. Additionally, the accidents that required hospitalization or were labeled as “serious” presented longer standard durations in all specifications. When we included individuals’ occupation, longer standard durations were found among skilled agriculture and fishing workers and machine operators (Specifications II & IV). Including sex in our frontier led men to experience slightly shorter standard sick leave durations compared with women (Specifications III & IV).

Once the standard durations were obtained for each individual, we calculated the estimated efficiency (EF). To do this, we used the quotient between the actual durations and those that would be expected because of medical or physiological factors presented in the Methods section. Thus, we can detect different behaviors between men and women globally and for different compensation levels.

Before obtaining the EF, we checked that all efficiency differences between men and women were statistically significant (see Table 3). We tested whether these differences were different from zero in all specifications and percentile levels and whether they were positive or negative, that is, “favoring” women or men. Analyzing the estimations for the whole distribution, we observed that women were more inefficient in Specifications I and II and more efficient in Specifications III and IV. In all cases, the test accepted the null hypothesis of different means for any significance level. Indeed, it was accepted in the first two specifications that the difference favors women, whereas in Specifications III and IV, the difference favors men. When considering compensation distribution, conclusions were not so homogeneous. In this case, we only considered Models I and III, given that the inclusion of occupational controls did not change the results. In Specification I, we found that men were more efficient if the compensation percentile was below 90. Between percentiles 90 and 95, there were no significant differences, and above percentile 95, women were more efficient. However, when we included the variable sex in the model (Specification III), women were always more efficient, although in the lower part of the distribution, this was significant at 5%.

**Table 3:** Equality of means hypothesis test for the estimated efficiency by sex

diff = mean(female) − mean(male)					
Specification	t-statistic	Ha: diff < 0	Ha: diff! = 0		Ha: diff > 0
I	*10.6149*	1.0000	0.0000		0.0000
II	*8.3480*	1.0000	0.0000		0.0000
III	− *17.2089*	0.0000	0.0000		1.0000
IV	− *17.1598*	0.0000	0.0000		1.0000
*Percentile (Specification I)*					
p0–p5	*5.2331*	1.0000	0.0000		0.0000
p5–p10	*4.7581*	1.0000	0.0000		0.0000
p10–p25	*5.8384*	1.0000	0.0000		0.0000
p25–p50	*5.8112*	1.0000	0.0000		0.0000
p50–p75	*9.0319*	1.0000	0.0000		0.0000
p75–p90	*3.0693*	0.9989	0.0021		0.0011
p90–p95	*0.0635*	0.5253	0.9493		0.4747
p95–p100	− *6.0533*	0.0000	0.0000		1.0000
*Percentile (Specification III)*					
p0–p5	− *1.7876*	0.0369	0.0738		0.9631
p5–p10	− *2.2839*	0.0112	0.0224		0.9888
p10–p25	− *5.7707*	0.0000	0.0000		1.0000
p25–p50	− *7.4457*	0.0000	0.0000		1.0000
p50–p75	− *4.3339*	0.0000	0.0000		1.0000
p75–p90	− *6.9604*	0.0000	0.0000		1.0000
p90–p95	− *5.7072*	0.0000	0.0000		1.0000
p95–p100	− *11.7147*	0.0000	0.0000		1.0000

Ho: diff = 0

We only included Specifications I and III because the occupational controls did not change the results

Source: Own elaboration

Once the existence of the significant differences was established, the EFs for males and females by compensation percentiles were obtained, as shown in Table 4 (and Figs. 7 and 8 in the ″Appendix”). In addition to performing the analysis for the whole sample of accidents, we also conducted all estimations only including injures that were “hard to diagnose,” that is, strains and sprains. This distinction was made because according to moral hazard literature, in this type of injury where the recovery period is not clearly determined, the existence of opportunistic behaviors among injured workers is common.

**Table 4:** Estimated efficiency by specification, percentile of the compensation, type of injury and sex

	Not including sex in the frontier								Including sex in the frontier							
	Specification I				Specification II				Specification III				Specification IV			
Percentiles	Total		HtD		Total		HtD		Total		HtD		Total		HtD	
	F	M	F	M	F	M	F	M	F	M	F	M	F	M	F	M
p0–p5	2.035	2.005	2.033	2.004	2.034	2.006	2.033	2.004	2.009	2.019	2.009	2.019	2.009	2.020	2.009	2.020
p5–p10	2.039	2.012	2.051	2.016	2.038	2.012	2.050	2.017	2.014	2.027	2.027	2.032	2.013	2.026	2.026	2.032
p10–p25	2.036	2.016	2.032	2.014	2.033	2.017	2.030	2.015	2.010	2.030	2.008	2.029	2.009	2.030	2.007	2.030
p25–p50	2.045	2.028	2.044	2.039	2.045	2.032	2.044	2.044	2.020	2.042	2.020	2.056	2.020	2.044	2.021	2.057
p50–p75	2.052	2.025	2.044	2.024	2.052	2.029	2.044	2.028	2.026	2.039	2.020	2.040	2.028	2.041	2.021	2.041
p75–p90	2.040	2.028	2.020	2.026	2.040	2.031	2.020	2.028	2.016	2.043	1.997	2.042	2.019	2.044	2.001	2.043
p90–p95	2.038	2.037	2.025	2.044	2.036	2.036	2.023	2.042	2.013	2.053	2.002	2.061	2.018	2.052	2.007	2.060
p95–p100	2.028	2.069	2.000	2.082	2.027	2.067	2.000	2.080	2.004	2.086	1.978	2.100	2.011	2.087	1.985	2.100

HtD refers to a frontier estimation with only “hard to diagnose” injuries

Source: Own elaboration based on SAW data

In general, the results show that both sexes extended their recovery periods longer across all percentile levels (i.e., *EF* > *1*). The first two specifications that did not include sex in the frontier estimation indicate that women extended their sick leave duration longer compared with men across all percentile levels, except for the highest percentile level. This fact was reinforced among injuries hard to diagnose, where men extended their recovery periods longer from percentile 90 compared with women. This behavior was not detected when including sex in our frontier (Specifications III & IV). In these cases, men extended their recovery periods longer across all percentile levels. This is because women have longer standard durations.

**Fig. 3:**
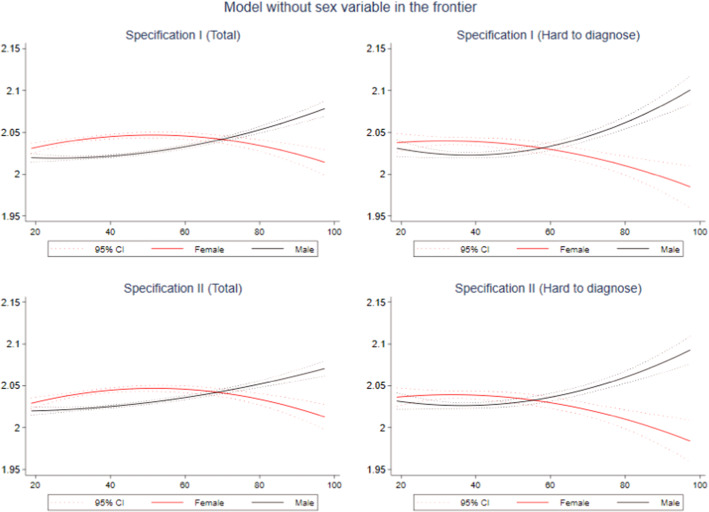
Quadratic prediction of efficiency using compensation by sex and type of injury: Specifications I and II. *Notes*: Dotted lines represent 95% confidence intervals (CIs). Source: Own elaboration based on SAW data

**Fig. 4:**
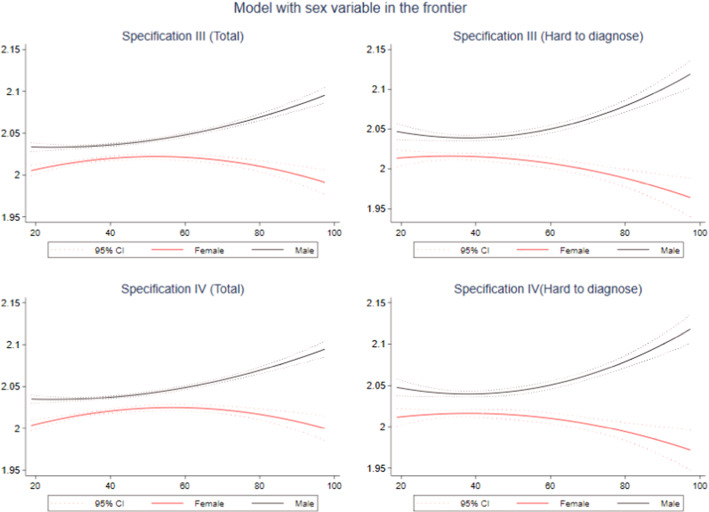
Quadratic prediction of efficiency using compensation by sex and type of injury: Specifications III and IV. *Notes*: Dotted lines represent 95% confidence intervals (CIs). Source: Own elaboration based on SAW data

To complete the analysis, Figs. 3 and 4 present predictions of the efficiency for the whole compensation distribution (for the total accidents and for the hard-to-diagnose groups). These predictions were obtained by regressing the estimated efficiency on compensation and compensation squared as can be seen in the following expression:

5 $${\text{EF}}_{{\rm i}} = {\beta}_{0}+ {\beta}_{1}{{\text{C}}}_{{\rm i}}+{\beta}_{2}{{\text{C}}}_{{\rm i}}^{2},$$

where ${\text{EF}}_{{\rm i}}$ is the efficiency measure obtained for an individual ${\text{i}}$ from Eq. 5, ${\text{C}}$ is the variable measuring the received compensation by the injured worker, and ${\beta}_{{\rm i}}$ are the coefficients to be estimated. The quadratic specification was chosen to detect nonlinearities because it fits the inefficiency patterns presented in Figs. 7 and 8 in the ″Appendix”. These predictions allow us to detect the point of the compensation distribution at which individuals become more (or less) inefficient than others. It is also possible to identify for which compensation these individuals change their behavior, from increasing to reducing the efficiency, or vice versa.

Table 6 in the ″Appendix” provides detailed information of Figs. 3 and 4, namely, coefficients of the squared estimations, precise compensations where the maximum and minimum inefficiencies reached (depending on whether the quadratic fitting provided a concave or a convex function), and the cut points between both distributions when available.

When not including sex in our frontier and considering the total of accidents (Fig. 3), inefficiency in men increases as compensation is higher in both specifications (I and II). However, inefficiency in women only increases until compensation reaches values close to EUR 55. From that point, inefficiency starts decreasing until it takes values lower than those of men, specifically when compensation reaches over EUR 70. In cases of hard-to-diagnose accidents (right graphs of Fig. 3), the situation slightly changes. Men only increase their inefficiency when compensation is higher than EUR 35, whereas in women, it starts decreasing from almost the beginning of the compensation distribution (approximately EUR 38 in the Specification I and EUR 39 in the Specification II). Consequently, inefficiency in women is below that in men from compensations close to EUR 60.

When we include sex in our frontier, we also observed different behavioral patterns between men and women (Fig. 4). Combining all accidents together, inefficiency in men presents a continuous growth, which is accelerated as compensation increases. On the contrary, women only increase their inefficiency until the compensation reaches values between EUR 55 and EUR 60. From that point, their inefficiency starts to decrease. If we only consider hard-to-diagnose accidents (right graphs of Fig. 4), men only reduce their inefficiency until the compensation reaches values of EUR 36, and from that point, it starts increasing significantly. After a certain stability in lower compensation levels, we observe a reduction in the inefficiency levels of women at higher compensations.

## Robustness analysis

To check the robustness of our results, we repeated the analysis for two concrete groups of injured workers—a specific part of the service sector workers (“Other services” sector specified in Table 1) and the most qualified ones.

The “other services” sector covers almost one million accidents that are more evenly distributed between men and women and between white-collar and blue-collar workers. These activities include workers in public administration, education, health, or domestic services. In general, these are non-male-dominated jobs where the gender bias in occupational accidents is corrected. The aim is to test whether the results obtained in the empirical section are conditioned by an asymmetric distribution of accidents highly concentrated in certain occupations, e.g., men develop most manual jobs.

**Fig. 5:**
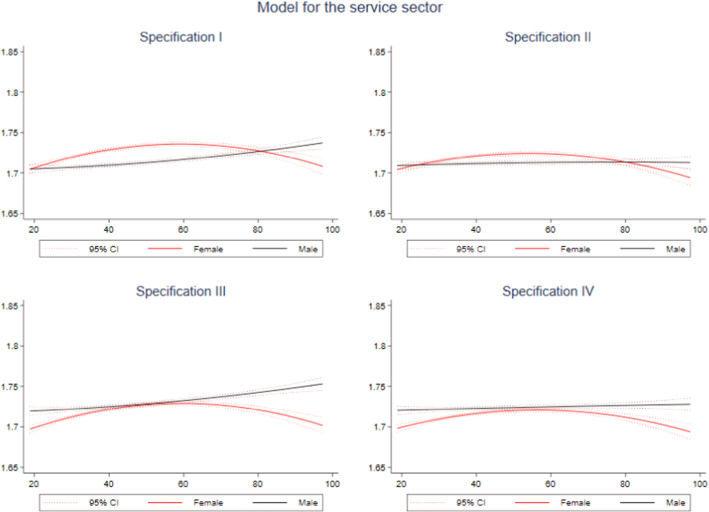
Quadratic prediction of EF in the service sector using compensation by sex. *Notes*: Dotted lines represent 95% confidence intervals. Source: Author’s own based on SAW data

The estimated coefficients are similar in sign and magnitude to those obtained in the total regression. However, when calculating the error terms, we observe a low contribution of inefficiency for both men and women. Figure 5 shows the results of the efficiency prediction by compensation distribution and workers’ sex for the four analyzed models. In the case of specifications I and II, those injured women at the lowest distribution scale present high inefficiency, corroborating our main results. The observed behavior change in the high levels of the compensation distribution is still plausible—the inefficiency is lower among women who receive compensations above EUR80 per day.

Secondly, we conducted another robustness analysis focusing on the most qualified workers. The prevalence of injuries differs across occupations, and the glass ceiling theory is associated with high-responsibility positions. We thus conducted additional estimations removing from our sample low-qualified workers.

**Fig. 6:**
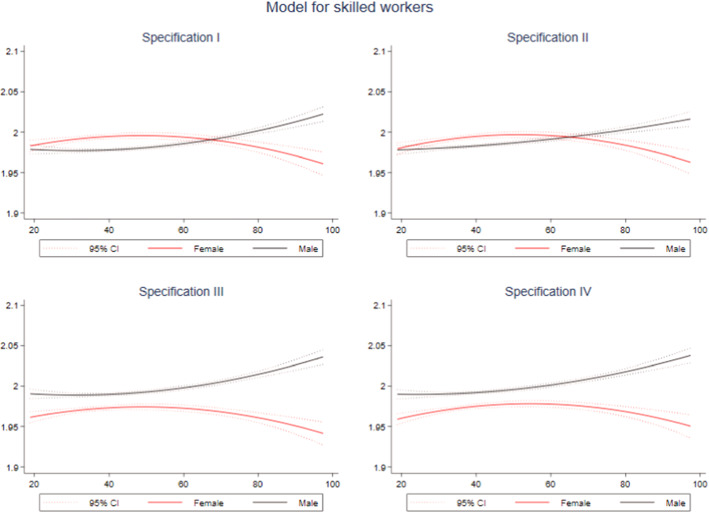
Quadratic prediction of EF for skilled workers using compensation by sex. Notes: Dotted lines represent 95% confidence intervals. Source: Author’s own based on SAW data

**Fig. 7:**
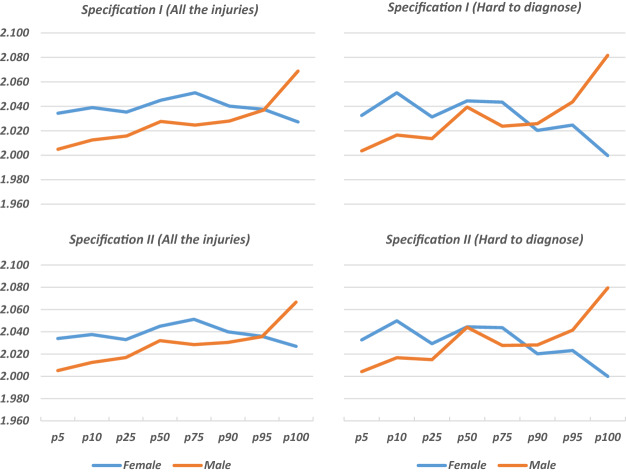
Estimated EF by sex, percentile of the compensation, and type of injury: specifications without sex in the frontier. Source: Own elaboration based on SAW data

**Fig. 8:**
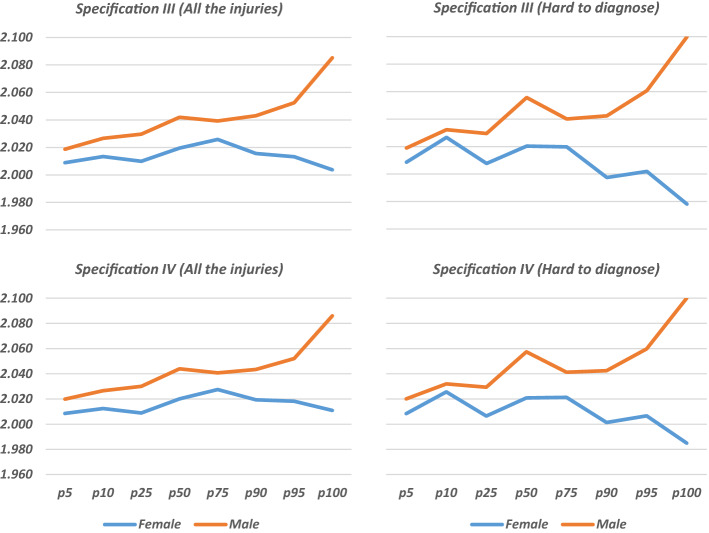
Estimated EF by sex, percentile of the compensation, and type of injury: specifications with sex in the frontier. Source: Own elaboration based on SAW data

As can be seen in Fig. 6, results back the patterns observed in our previous estimations, i.e., when considering the total of accidents and the specific part of the services sector. In specifications I and II, where not including the sex variable as a control, we again observe better behavior among men at low compensation levels and among women when the compensation is above EUR65. When including the sex variable (specifications III & IV), the inefficiency degree is higher among males than females in all cases, especially at high compensation levels. As with the service sector sample, we observe a reduction in the inefficiency of these workers.

This robustness analysis allows us to conclude that the observed gender differences in behavior are not related to a higher concentration of accidents among males or to a higher amount of accidents affecting low-skilled workers.

## Policy implications

There exist two areas in which this study may contribute to designing well-targeted policy interventions. On the one hand, our results shed light on the significant issues concerning the public health domain. On the other hand, labor policymakers may also find some of the outcomes obtained insightful.

From the public health perspective, a first outcome that can be useful is the division within sick leave duration between days associated with pure physiological determinants and days derived from behavioral aspects. This enables policymakers to address the question properly. For instance, if in a specific group the authentic problem is the opportunistic behavior that extends the sick leave excessively, it would be a mistake to devote a great proportion of the safety budget to develop group-specific safety-enhancing measures to avoid serious injuries. Vice versa, if the problem relies on the lack of preventive instruments and procedures, implementing a strict monitoring strategy to avoid absenteeism would not be appropriate.

Moreover, in this study, the econometric model estimated allows policymakers and insurance managers to compute what a standard duration (i.e., that duration depending only on physiological factors) should be for a set of explanatory variables. With this, it is possible to identify problematic situations from a moral hazard standpoint at the individual level. Hence, it is straightforward to pinpoint those people that may be behaving more opportunistically and take action to address the issue in an informed manner.

From a gender/sex perspective, our estimates show that there are significant differences between women and men regarding sick leave duration, thus posing important policy questions. As our estimates suggest, if women have worse health status in the workplace, gender-specific measures from a public health point of view should be implemented to tackle this issue.

Second, there also exist distinct behavioral responses between genders. Although a simple comparison between women and men regarding “behavioral” days off within sick leave shows no significant differences, this observation is hiding a twofold divergent pattern when considering the income distribution ladder. Opportunistic behavior is higher at the top of the wage distribution in case of men and for low wage levels in case of women. From a policy standpoint, these results suggest devoting more financial resources to screening activities to check low-income women and high-income men as well.

Our results also enable the implementation of fine-tuning policies, as it is possible to accurately determine some relevant thresholds. For instance, it is feasible to compute the wage level at which the opportunistic behavior in men stops decreasing and starts increasing (i.e., the arg min. value). Moreover, the arg max. value of the wage for women can be calculated (i.e., the wage at which the opportunistic behavior in women stops increasing and starts decreasing). Finally, policymakers and insurance managers can assess which group performs more opportunistic behavior at different wage levels, which, in turn, allows them to develop targeted monitoring policies enhancing efficiency.

From a labor relations point of view, our results put forward a response in the behavior of women as a result of the institutional and social barriers in the labor market. Two theories have been popularized in the last decades regarding the career advancement in women: the sticky floor and the glass ceiling hypotheses.

The sticky floor hypothesis posits that there is a discriminatory employment pattern that retains workers, mainly women, in lower ranks of the job scale, which leads to low wages, low job mobility, and invisible barriers to career advancement. This fact could demotivate low-wage women, preventing them from exerting high effort levels, and this is what we precisely observe in our data. The policy prescription would be to raise expectations relating to job promotions to encourage women and to achieve more work-committed low-wage female employees. The main institutional device to achieve this goal is to foster permanent employment among women. Fixed-term labor contracts are widespread within the Spanish labor market, disproportionately affecting women. This type of hiring is associated with low wages and poor career advancement perspectives. Thus, reducing overall temporary labor contracts, particularly among women, should help solve this problem.

The glass ceiling hypothesis postulates that there exists an unacknowledged barrier to advance in a profession, particularly affecting women and minority groups. It is often considered that the glass ceiling is particularly important in the higher ranks of the job scale. In this case, women might be exerting an additional effort (i.e., carrying out a “presentist behavior”) so as to tear down this discriminatory barrier. In other words, in an attempt to break the glass ceiling, women might be striving too hard, which is not efficient, creating room for policy action to fix this market failure. The gender gap in the behavioral component of the sick leave at the top of the wage distribution that we observe in our estimates seems to point toward that direction, supporting the existence of a significant glass ceiling in the Spanish labor market. The policy prescription would be to implement antidiscrimination measures targeted at workers at the top of the wage distribution.

## Conclusions

The gender/sex gap in the duration of sick leave has been traditionally studied by analyzing ex-post biological differences between men and women. As behavioral responses are an important factor that may explain this gap, no prior studies have analyzed their role in explaining these differences. This study fills this gap in the literature by adopting a methodology scarcely used in this field of research, that is, the frontier analysis technique, which allows us to split sick absence days into two types of days—those that are exclusively related to biological characteristics and those that are derived from behavioral reasons.

We found that women present longer standard durations, that is, more days purely attached to physiological characteristics, compared with men, thus showing less opportunistic behaviors. However, when we assumed that there are no biological differences between men and women, behavioral differences were negligible.

Gender differences were more plausible when considering the amount of sick leave benefit. If we assume that men and women recover at the same rate from the same injury (i.e., without including the sex variable in our frontier), we found that women are more inefficient at lower levels of income, whereas in case of men, inefficiency is more pronounced at higher wage levels. However, when considering biological differences (i.e., including the sex variable in our frontier, and interaction terms between this variable and each type of injury), we found that women were more efficient than men across all the income distribution, especially at higher levels of income.

Therefore, we provide evidence that it is essential to consider biological and behavioral characteristics when analyzing the gender/sex gap in the duration of sick leaves as it gives a reasonable image of what these differences really hide.

## Appendix

See Tables 5, 6 and 7.

**Table 5:** Daily amount of sick leave compensation by year in Spain

	Contribution base		75% Contribution base		Deflator
	Maxim	Minim	Maxim	Minim	
2011	107.67	24.94	80.75	18.71	0.9708
2012	108.75	24.94	81.56	18.71	0.9946
2013	114.19	25.10	85.64	18.83	1.0086
2014	119.90	25.10	89.93	18.83	1.0071
2015	120.20	25.22	90.15	18.92	1.0020
2016	121.40	25.48	91.05	19.11	1.0000
2017	125.04	27.52	93.78	20.64	1.0196
2018	125.04	28.62	93.78	21.47	1.0366
2019	135.67	35.00	101.75	26.25	1.0439

Source: Own elaboration based on data of Social Security and Spanish Statistical Office

**Table 6:** Estimated coefficients, minimum, maximum, and cut points of the efficiency quadratic fit on the compensation distribution (Figs. 3 and 4)

	Male				Female				Cut point
Specif	${\beta}_{0}$	${\beta}_{1}$	${\beta}_{2}$	Min	${\beta}_{0}$	${\beta}_{1}$	${\beta}_{2}$	Max	
I	2.0248	− 0.00017	0.00001	13.9	2.0001	0.00179	− 0.00002	54.5	71.27
I (HtD)	2.0510	− 0.00158	0.00002	36.4	2.0197	0.00104	− 0.00001	37.9	59.00
II	2.0161	− 0.00004	0.00001	3.0	1.9964	0.00191	− 0.00002	54.8	69.83
II (HtD)	2.0464	− 0.00121	0.00002	34.5	2.0170	0.00113	− 0.00001	39.1	57.07
III	2.0351	− 0.00042	0.00001	19.3	1.9746	0.00178	− 0.00002	55.5	None
III (HtD)	2.0667	− 0.00158	0.00002	36.0	1.9953	0.00104	− 0.00001	38.8	None
IV	2.0361	− 0.00040	0.00001	19.0	1.9703	0.00191	− 0.00002	59.3	None
IV (HtD)	2.0673	− 0.00156	0.00002	36.2	1.9910	0.00118	− 0.00001	43.6	None

In case of men, we included the minimum value as the quadratic fit was convex (opening up parabola)

In case of women, we included the maximum value as the quadratic fit was concave (opening down parabola)

We only include the cut point shown in the graphs

HtD refers to a frontier estimation with only “hard to diagnose” injuries

**Table 7:** Frontier estimations of the logarithm of the sick leave duration with gender differences in injuries. Source: Own elaboration based on SAW data

	Not including sex variable			
	Specification I			
Log duration	Coef	P > z	Coef	P > z
Injury			Injury * male	
Unspecified injuries (ref.)			− **0.106**	0.000
Superficial injuries	− 0.156	0.000	− **0.062**	0.000
Other injuries	− 0.189	0.000	0.033	0.000
Fractures	1.067	0.000	− **0.045**	0.000
Strains	0.030	0.000	− **0.092**	0.000
Dislocations	0.068	0.000	− **0.059**	0.000
Sprains	0.028	0.000	− **0.075**	0.000
Traumatic amputation	0.837	0.000	0.196	0.000
Concussion	0.018	0.022	− **0.028**	0.000
Burns	− 0.437	0.000	0.211	0.000
Poisoning	− 0.421	0.000	− **0.083**	0.001
Choking	− 0.604	0.000	− **0.195**	0.000
Noise, heat, radiation	− 0.182	0.000	− **0.070**	0.019
Psychological trauma	0.045	0.005	− **0.140**	0.000
Multiple injuries	0.112	0.000	0.056	0.000
Heart attack	0.784	0.000	0.147	0.000
Part of the body	***Yes***			

	Coef	P > z
Hospital care	0.185*	0.000
hospitalization	0.612	0.000
Serious	1.030	0.000
Recurrence	0.399	0.000
Age	0.009	0.000
Age squared	0.000	0.000
Constant	1.860	0.000
sigma_v	0.854	
sigma_u	0.519	
sigma2	0.999	
Lambda	0.608	
Data	3,916,249	

See Figs. 7 and 8.

## Footnotes

1 Health predicts absenteeism for everybody; however, the population is heterogeneous in engaging in opportunistic behaviors (Suárez & Muñiz, 2018).

2 In 2020, women spent an average of 263.4 min per day in unpaid work, whereas men spent 136.5 min per day in unpaid work in Organisation for Economic Co-operation and Development countries. In Spain, women spent an average of 289.1 min per day in unpaid work, whereas men spent an average of 145.9 min per day in unpaid work (OECD, 2021).

3 According to the Global Gender Gap Report, the percentage of women participating in the labor market was 69.2, whereas in the case of men, this figure increased up to 79.1% (World Economic Forum, 2021).

4 These types of jobs include temporary, part-time or per-day contracts, and contingent jobs.

5 These include anterior cruciate ligament tears, multidirectional shoulder instability, and ankle instability or osteoporosis.

6 To calculate the maximum duration of the TI and its possible extension, the relapse and observation periods are considered. A relapse period would be included in the same TI when this happens during the next 180 days from the previous recovery, and the new medical sick leave is due to the same or similar injury.

7 There are also included all those working accidents that occurred on worker’s way home and to work, or because of executing a union position or any work-related task (although not related to his/her main position, but demanded by the employer), and those injuries previously endured by the worker if they have been exacerbated by the working accident.

8 Table 5 in the ″Appendix” includes the maximum and minimum values of the daily contribution base and the inflation index used to adjust the workers compensation variable.

9 Such behavior has often been reported in the literature addressing work absenteeism (e.g., Zhang et al., 2017) and in the literature exploring issues of moral hazard linked to work accident insurance (e.g., Martin-Roman and Moral 2016; Martín-Román and Moral 2017).

10 Within the methodological framework of this study, this lower frontier is linked to what is termed as the cost frontier in the literature.

11 Not being able to estimate the value of the variances separately prevents one from performing the necessary tests to validate the existence of inefficiency.

12 Men had long sick leave durations when they suffered from “other injuries”; burns, multiple injuries, traumatic amputation or heart attacks.

13 Because the test lies on the boundary of the parameter space of ${\sigma}_{\text{u}}^{2}$, the standard likelihood-ratio test is not valid, and a one-sided generalized likelihood-ratio test must be constructed (Gutierrez et al., 2001).
